# Outcomes of Mulligan Concept Applications in Obese Individuals with Chronic Mechanical Low Back Pain: A Randomized Controlled Trial

**DOI:** 10.3390/life14060754

**Published:** 2024-06-13

**Authors:** Muhammed Safa Cankaya, Omer Osman Pala

**Affiliations:** 1 Vocational School of Health Services, Erzincan Binali Yıldırım University, Erzincan 24002, Turkey; safa.cankaya@erzincan.edu.tr; 2 Department of Physiotherapy and Rehabilitation, Faculty of Health Sciences, Bolu Abant İzzet Baysal University, Bolu 14030, Turkey

**Keywords:** musculoskeletal manipulations, obesity/diagnosis, pain measurement, randomized controlled trials as topic, range of motion, articular

## Abstract

Background: Various treatment modalities have been employed for mechanical low back pain (MLBP), but evidence of their efficacy varies greatly. Objectıve: This randomized controlled trial aimed to assess the outcomes of Mulligan concept applications, including sustained natural apophyseal glides (SNAGS) and natural apophyseal glides (NAGS), in obese patients with MLBP. Methods: The study, conducted between January 2021 and June 2022 at a tertiary hospital, involved randomizing patients into two groups. Both groups underwent six sessions of stretching and strengthening exercises every other day. The Mulligan group received additional intervention with SNAG and NAGS techniques. Measurements were made regarding the Visual Analog Scale (VAS) score, Oswestry Disability Index (ODI) score and range of motion (ROM) for the patients’ MLBP level. Results: Post-interventions, both groups exhibited positive changes in flexion ROM, extension ROM, right and left rotation ROM, right and left lateral flexion ROM, VAS score, and ODI score compared to pre-intervention (*p* < 0.001 for both groups and variables). The Mulligan group showed a higher increase in ROM and a more significant decrease in VAS and ODI scores. Conclusıons: Mulligan mobilization techniques prove significantly beneficial for enhancing ROM in all directions, reducing pain levels, and alleviating disability in obese individuals with MLBP.

## 1. Introduction

Today, low back pain is a leading cause of disability worldwide. The lifetime burden of disability due to low back pain increased by more than 60% during the last four decades, likely driven by population growth and ageing [1]. Only a small proportion of cases with low back pain can be directly linked to a pathological cause. Low back pain (lumbago) is usually nonspecific or mechanical. Mechanical low back pain can originate from the spine, intervertebral discs or surrounding soft tissues [2]. Other factors associated with low back pain include smoking, health status, comorbidities (asthma, diabetes, headache, osteoarthritis and osteoporosis), physical workload, low physical activity, and mental health disorders [3].

Being overweight or obese increases the risk of low back pain due to excessive load on the joints and changes in body composition [4,5,6,7,8]. According to the most widely accepted definitions, a person is ‘overweight’ when they have a body mass index (BMI) of >25 and ‘obese’ with a BMI of >30 [9]. A high BMI has a considerable impact on the lumbosacral joints. When examined percentage-wise, each unit of increase in body weight has been demonstrated to cause a two-fold increase in compressive force at the L5-S1 joint [10]. Another important factor worth considering is that adipose tissue in obese patients secretes a number of proinflammatory and anti-inflammatory cytokines. C-reactive protein (CRP), a proinflammatory marker of acute inflammation, has been linked to musculoskeletal pain. Prostaglandin E2, another noteworthy inflammatory marker, is involved in fever, pain sensation, and inflammation associated with low back pain [3,11].

There are various treatment modalities for mechanical low back pain, including manipulative techniques, which have been reported to provide favourable results [2]. Manual therapy techniques, such as the Mulligan technique, Maitland mobilization, Kaltenborn technique, and active release, are among the main strategies used in physiotherapy to manage neuromuscular pain, including low back pain [12]. Positional errors in joints, which may result from injury or prolonged/perpetual force can cause pain and restricted range of motion (ROM). In such cases, the Mulligan technique, a manual therapy approach, may be a crucial solution. This technique involves mobilization to normalize both arthrokinematics and osteokinematics of the joint, and it is particularly valuable in correcting postural errors [13]. Indeed, previous studies have reported positive outcomes with the Mulligan technique in terms of pain, disability and ROM in patients with low back pain [12,14]. However, published research on obese individuals is limited, who experience a substantially higher risk of low back pain compared to those with normal weight.

In this study, we aimed to evaluate the results of utilizing Mulligan techniques (Sustained natural apophyseal glides; SNAGS and Natural Apophyseal Glides; NAGS) on pain, disability and ROM in obese individuals with chronic mechanical low back pain.

## 2. Material and Methods Study Design

This randomized controlled trial was conducted between November 2021 and April 2022 at Erzincan Binali Yıldırım University, Mengücek Gazi Training and Research Hospital.

Ethics committee approval was obtained from Clinical Research Ethics Committee of Erzincan Binali Yıldırım University for the study (Decision date: 25 October 2021, decision no: #2021-11/04). The study was registered at www.clinicaltrials.gov under NCT06201286. All stages of the study were conducted in accordance with the Declaration of Helsinki. Following comprehensive information provided to the patients in the study group about the study’s purpose and scope, those who consented to participate signed the written consent form.

### 2.1. Sample Size and Grouping

**Figure 1:**
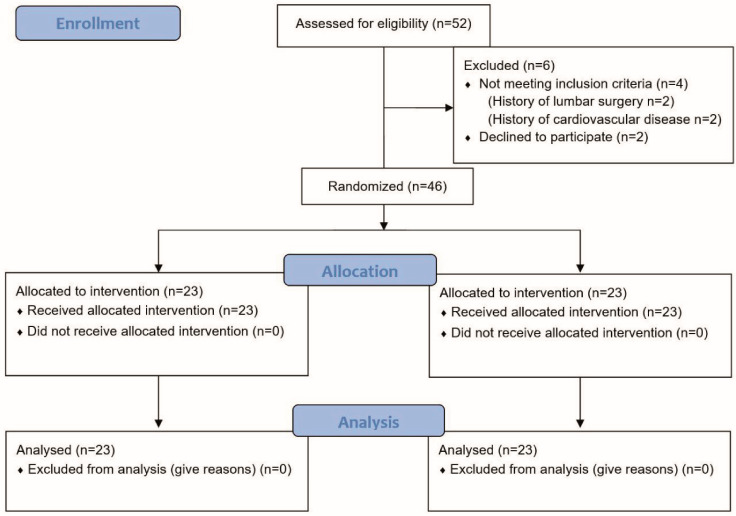
Flow diagram of the study.

Power analysis based on prior data demonstrated that including 38 patients would achieve a power (1-beta) of 0.80 at a significance level (alpha) of 0.05. Power analysis was performed using the F test and ANCOVA tabs of G*Power Version 3.1.9.4 software, while the effect size (0.75) was obtained from the study by Hidalgo et al. [15]. The inclusion criteria were having mechanical low back pain for at least three months (diagnosed by family doctor), being between 18 and 50 years of age, and having a BMI between 30.00 and 39.99. Patients with a history of surgical intervention in the lumbar region, those with a diagnosis of cardiovascular disease or pregnant women, those with exercise intolerance for any reason, and subjects who refused to participate in the study were excluded. The 46 study subjects were randomly divided into two groups (Figure 1). Stratified randomization was applied to ensure equal numbers of men and women in the groups. An online randomisation tool “GraphPad” was used for treatment assignment (Mulligan group versus control group) [16].

### 2.2. Interventions

Both the Mulligan group and the control group were subjected to an approximately 30 min, 6-session exercise program. A one-day gap separated each session, and the entire program spanned 2 weeks. Both groups participated in supervised stretching and strengthening exercises. Exclusive to the Mulligan group, they received SNAG and NAGS exercises as part of the Mulligan concept applications, utilizing tools like the Mulligan belt, sponge, and stretcher. Meanwhile, traditional physical therapy methods and strengthening exercises were conducted using a mat, weights, and a Swiss ball.

### 2.3. Stretching Exercises

Stretching exercises were directed at the erector spinae and intertransverse lumborum muscles within the extensor muscle groups. This regimen comprised three sets of 10 repetitions, with each stretch lasting 15–30 s. Lumbar region extensor stretches involved pulling the legs to the chest in the supine position, while intertransverse lumborum muscle stretches included pulling the knees to the abdomen and incorporating right-left rotation in the same supine position [17].

### 2.4. Strengthening Exercises

Strength training targeted the rectus abdominis, internal oblique, external oblique, and transversus abdominis muscles within the flexor muscle groups, featuring three sets of 10 repetitions. Additionally, Swiss ball exercises were incorporated to strengthen the multifidus muscle. For the multifidus muscle, patients were seated on a Swiss ball and engaged in strengthening movements by lifting the head upward, imagining an object above without dismounting from the Swiss ball [17].

### 2.5. Mulligan Mobilization Techniques

Mulligan mobilization techniques were administered by a physiotherapist with Mulligan Concept A-B module training, involving three sets of 10 repetitions and 15–20 s of rest between sets. Accurate application of the Mulligan technique requires joint positioning under load, ensuring painless pressure at the terminal stage of movement, achieving pain-free movement post-application, and utilizing it at the maximum painless range of motion. Techniques such as Sustained Natural Apophyseal Glides (SNAGS) and Natural Apophyseal Glides (NAGS) were applied to the spinous process of each lumbar vertebra. Participants experiencing severe pain in the standing position received mobilization in the sitting position. In the standing position, the physiotherapist positioned themselves beside the patient, stabilizing them by gripping the abdomen with one arm. Using a sponge to prevent slippage, the NAGS technique was initially applied to each lumbar level with the thenar region of the other hand.

### 2.6. Sustained Natural Apophyseal Glides (SNAGS)

The SNAG method is a lumbar vertebral mobilization technique that smoothly slides towards the problematic facet joint in a weight-bearing position, promoting natural movement at the end of the joint opening. Widely utilized for vertebral column mobilization due to its minimal contraindications, the SNAG method combines active and passive elements. Pressure is applied precisely at the endpoint of the movement, targeting either the facet joint directly (unilateral technique) or the vertebral spinous processes. In patients who could stand, these movements were performed in the standing position, but in the presence of problems such as excess weight, these movements were performed in the sitting position.

### 2.7. Natural Apophyseal Glides (NAGS)

The NAGS technique, often referred to as passive oscillatory movements, is a mobilization approach that entails a smooth sliding motion along the surface of the troublesome facet joint in the low back vertebrae. This method is implemented when the body is bearing weight.

### 2.8. Data Collection and Measurements

The patients’ sociodemographic features, Visual Analogue Scale (VAS) score reflecting the extent of mechanical low back pain, Oswestry Disability Index (ODI) score, and comprehensive data on range of motion were documented. Initial measurements were conducted on day 1, right before the initial intervention. Subsequent measurements were made at two weeks, immediately after the last intervention in session 6 [18]. In the study, VAS score, ODI score and ROM values were accepted as the primary outcome measures.

### 2.9. Range of Motion (ROM)

DrGoniometer^®^ was used for lumbar ROM measurements, which is established to be a reliable approach for ROM measurements. Active flexion, extension, lateral flexion and rotation of the lumbar region were measured while the subjects were in the sitting position.

### 2.10. Visual Analogue Scale (VAS)

VAS was used to assess the severity of mechanical low back pain. Individuals were asked to mark the pain they felt on a 10 cm paper-strip VAS scale. The patients were given clarifying information about the use of VAS. Briefly, on the VAS, 0 was defined to indicate no pain, while 10 indicated the greatest pain in their life.

### 2.11. Oswestry Disability Index (ODI)

The disability levels of the patients attributed to low back pain were evaluated with the Oswestry Disability Index (ODI), the Turkish validity and reliability of which was performed by Yakut et al. [19]. The ODI comprises 10 items evaluating pain severity, personal care, lifting–carrying, walking, sitting, standing, sleeping, social life, travelling, and pain change. Each item offers six response options, scored between 0 and 5. The cumulative scores from all items determine the ODI score, calculated using the formula ODI score = (patient’s score/maximum possible score = 50) × 100. The resulting value indicates the percentage of the patient’s disability level (0–20% = low impact on daily life, 20–40% = mild limitation, 40–60% = substantial limitation, 60–80% = severe limitation, 80–100% = significant impairment or bedridden).

### 2.12. Statistical Analysis

A significance level of *p* < 0.05 was adopted and analyses were conducted with the SPSS version 25.0 software (IBM Corp., Armonk, NY, USA). The normal distribution of variables was assessed using the Shapiro–Wilk test. Descriptive statistics, including mean ± standard deviation for continuous variables and frequency (percentage) for categorical variables, were presented. Between-group comparisons for normally distributed variables utilized the Student’s *t*-test, while the Mann–Whitney U test was employed for non-normally distributed variables. Categorical variables were compared using the chi-square test or Fisher’s exact test or Fisher–Freeman–Halton test. Two-way repeated measures analysis of variance (ANOVA) was used for normally distributed repeated measurements, and the Wilcoxon signed ranks test was applied for non-normally distributed repeated measurements. Additionally, post-treatment outcomes were analyzed using the analysis of covariance (ANCOVA) for normally distributed variables and Quade’s nonparametric ANCOVA for non-normally distributed variables with baseline measurements/scores as covariates. Partial eta squared (η^2^) effect sizes were calculated to evaluate the effect size. Effect sizes lower than 0.400 were accepted as small effects, between 0.400 and 0.799 were accepted as medium effects and effect sizes equal to or higher than 0.800 were accepted as large effects [20].

## 3. Results

The study included 46 participants, with 30 men (65.2%) and 16 women (34.8%), having a mean age of 37.35 ± 8.85 years (range 19–50). In the intervention groups, there were no losses after randomisation or adverse outcomes resulting from the interventions. There were no significant differences between the groups regarding age (*p =* 0.374) and gender (*p* = 1.000) distribution. Additionally, no significant differences were found in height (*p =* 0.819), weight (*p =* 0.752), BMI (*p =* 0.272), education status (*p =* 0.156), marital status (*p =* 0.336) and dominant status (*p* = 0.699). These similarities demonstrated the presence of a well-matched study population (Table 1).

No significant differences were found in dominant status (*p* = 0.699). Following the interventions, both groups exhibited significant improvements in flexion ROM, extension ROM, right rotation ROM, left rotation ROM, right lateral flexion ROM, left lateral flexion ROM, VAS score, and ODI score values compared to pre-intervention (*p* < 0.001 for each group, as shown in Table 2).

The group that underwent Mulligan exercises experienced a significantly greater improvement in flexion ROM (*p* < 0.001, η^2^ = 0.334), extension ROM (*p* < 0.001, η^2^ = 0.606), right rotation ROM (*p* < 0.001, η^2^ = 0.495), left rotation ROM (*p* < 0.001, η^2^ = 0.317), right lateral flexion ROM (*p* < 0.001, η^2^ = 0.536), and left lateral flexion ROM (*p* < 0.001, η^2^ = 0.442) compared to the control group (Table 2). According to these results, flexion ROM and left rotation ROM had small effects while extension ROM, right rotation ROM, right lateral flexion ROM and left lateral flexion ROM had medium effects.

**Figure 2:**
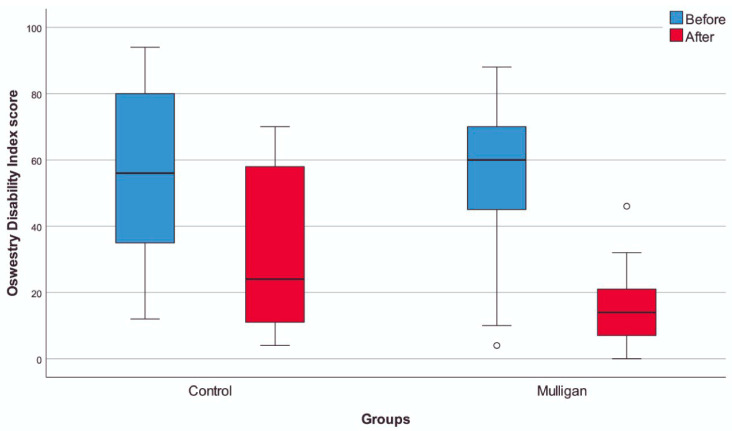
Oswestry Disability Index scores with regard to groups.

The VAS score exhibited a more substantial decrease in the group treated with the Mulligan mobilization technique compared to the control group (*p* < 0.001, η^2^ = 0.524). Additionally, the ODI score demonstrated a significantly greater improvement in the Mulligan group compared to the control group (*p* < 0.001, η^2^ = 0.441, Table 2, Figure 2). Both VAS and ODI scores had a medium effect.

## 4. Discussion

In this study, we aimed to compare the control group with the patients receiving Mulligan concept exercises in terms of pain, range of motion and functional status in obese patients with mechanical low back pain. Notably, the study group, which included Mulligan exercises, showed a significantly superior improvement in specific ROM directions such as flexion, extension, rotation and lateral flexion compared to the control group. The study group also experienced a more pronounced reduction in pain and a greater improvement in disability rate than the control group. These findings suggest that SNAGS and NAGS mobilisation may provide additional benefits and are effective compared to conventional interventions in the treatment of chronic mechanical low back pain in obese patients.

Obesity manifests as a comprehensive restriction in spinal movement. Obese individuals with chronic low back pain exhibit greater spinal limitation compared to those without low back pain. From a biomechanical perspective, obese individuals form a unique subgroup among chronic low back pain patients, indicating the need for tailored interventions involving specific management approaches [21]. Overweight or obese individuals with low back pain struggle with the combined physical challenges of physical activity and pain during their daily lives. With exercise programs, positive changes in various factors such as musculoskeletal pain, pain-related disability perception, functional ability, quality of life and body composition can be achieved in these individuals [22]. Obesity can potentially limit the impact of treatments for low back pain. In a study by Cuesta-Vargas et al., they assessed the outcomes of an 8-week physiotherapy program for chronic non-specific low back pain in both obese and non-obese individuals. The results indicated that, post-intervention, the non-obese group experienced significantly greater improvements in disability, physical aspects of quality of life, and overall quality of life compared to the obese group. Nevertheless, positive changes were noted in low back pain cases associated with obesity through physiotherapy interventions [23]. In a 4-week intervention using the SNAG technique for chronic mechanical low back pain, it was observed that lumbar range of motion significantly increased, and there were significant decreases in both VAS and ODI scores [24]. Kumar et al. found that a 4-week, 12-session program of Mulligan technique exercises resulted in significant improvements in ROM, VAS scores, and functional scores in individuals with chronic nonspecific low back pain [25]. In a similar study, SNAG mobilization was found to significantly enhance pain relief, functional independence, and ROM values following intervention [26]. Modified SNAGS has also been demonstrated to improve pain, function and lumbar flexion ROM values [27]. These treatments are also proven to improve short-term outcomes in terms of pain and function [15,28]. In the present study, the results of improvement in flexion ROM, extension ROM, right rotation ROM, left rotation ROM, right lateral flexion ROM, left lateral flexion ROM, low back pain VAS score and ODI values with SNAGS and NAGS support the results in the literature. In a meta-analysis of 47 randomized trials evaluating the effects of spinal manipulation techniques on chronic low back pain, it was reported that these treatments produced similar effects to standard treatments for chronic low back pain [29]. Compared to soft tissue mobilization, SNAGS are suggested to cause greater improvements in various outcome measures [30]. Khan et al. reported that both SNAGS and the Maitland technique improved pain, ROM and ODI scores; however, SNAGS values were again superior [31]. Similarly, Hussein et al. explained that SNAG caused more improvement in low back pain symptoms compared to the sham group. [32]. In the comparison of SNAGS versus myofascial release + strengthening exercises, Mulligan SNAGS had better short-term results, particularly for lumbar flexion ROM [33]. A two-week treatment plan for both the Mulligan and Maitland techniques revealed that pain reduction and functional improvement were better with the Mulligan approach [34].

In addition to significant advantages in various populations, a randomized controlled trial showed that 5 weeks of therapy improved functional characteristics, with the improvements lasting until the 6th month [35]. Another randomized controlled study reported notably greater improvements in SNAG recipients compared to those who only received strengthening exercises [13]. Similarly, SNAGS was found to lead to improvements in pain, flexion ROM, and functional status, within 3 weeks of lumbar treatment to a greater degree compared to conventional therapy, and it was evident that these effects were sustained up to 6 months [36]. The aforementioned results and the findings of randomized controlled trials in the field indicate that SNAGS is a very effective method in the management of low back pain. In the present study, in accordance with many results published in the literature, we found that the improvement in pain, ROM and disability was significantly greater in obese patients who were in the study group compared to controls. Although we did not evaluate the long-term trend of these positive effects, we believe that the use of SNAGS and NAGS mobilisation techniques in chronic mechanical low back pain can be effective in the short term as they provide benefits in improving symptoms and functional status. It is, therefore, evident that obese subjects also benefit from SNAGS and NAGS therapies, similar to non-obese populations, indicating that these readily available and established techniques can be successfully administered to overweight and obese individuals. Nonetheless, it is critical to note that interventions aimed at reducing BMI are also of utmost importance in such patients. In a meta-analysis of 10 studies (approximately 30,000 individuals), it was reported that being overweight or obese was strong risk factors for low back pain [37], establishing that BMI reduction remains an important approach to sustained relief of pain and functional disabilities in overweight or obese patients. The inclusion of mobilisation techniques in intervention research to reduce BMI may be beneficial in obese individuals at high risk of chronic meaknic low back pain.

The fact that the interventions were not applied over a longer period is a limitation of the study, especially since these exercises could also lead to weight loss in the long run. Another limitation is that the measurements for data collection were performed immediately after the intervention and at a single time point. Despite the fact that the majority of the literature agrees on the sustained effects of these therapies, it is evident that demonstrating these results in our population would have been an important contribution to the literature. It is also clear that the effectiveness of stretching, strengthening exercises and the administration of Mulligan techniques may vary based on the clinical experience of physiotherapists and the different issues that may arise with individuals with impaired mobility due to excess weight. Therefore, standardization of the therapeutic approach and Mulligan techniques will require preliminary studies to understand the needs of the specific patient. Comorbidities other than the factors examined in the study may have affected the measured variables, including data that were not collected. These could have biased the results and comparative findings despite the fact that randomization was performed for group selection. Nonetheless, this randomized controlled trial has remarkable results because it evaluated the results of Mulligan concept applications in low back pain, especially in obese individuals, which have had limited representation in the literature.

## 5. Conclusions

The key finding of this study emphasises the superior short-term efficacy of adding Mulligan’s SNAGS and NAGS techniques to stretching/strengthening exercises for the treatment of low back pain in obese patients, as demonstrated by pain relief, improved freedom of movement and improved functioning. To characterize these findings, determine therapeutic efficacy, and ascertain the long-term effectiveness of Mulligan techniques among individuals with obesity and low back pain, future research may benefit from population-based, longitudinal, and prospective studies.

## Figures and Tables

**Table 1:** Summary of demographics with regard to groups.

	Groups		
	Control (*n* = 23)	Mulligan (*n* = 23)	*p*
Age	36.17 ± 9.60	38.52 ± 8.06	0.374 ^†^
Sex			
Male	15 (65.22%)	15 (65.22%)	1.000 ^#^
Female	8 (34.78%)	8 (34.78%)	
Height, cm	171.87 ± 9.45	172.57 ± 11.02	0.819 ^†^
Weight, kg	98.30 ± 13.51	97.09 ± 12.41	0.752 ^†^
Body mass index, kg/m^2^	33.13 ± 1.87	32.51 ± 1.55	0.272 ^‡^
Education status			
Primary school	0 (0.00%)	0 (0.00%)	0.156 ^¶^
Secondary school	4 (17.39%)	6 (26.09%)	
High school	12 (52.17%)	6 (26.09%)	
University	7 (30.43%)	8 (34.78%)	
Postgraduate	0 (0.00%)	3 (13.04%)	
Marital status			
Married	18 (78.26%)	14 (60.87%)	0.336 ^#^
Single	5 (21.74%)	9 (39.13%)	
Dominant side			
Right	20 (86.96%)	18 (78.26%)	0.699 ^§^
Left	3 (13.04%)	5 (21.74%)	

Descriptive statistics were presented by using mean ± standard deviation continuous variables and frequency (percentage) for categorical variables. ^†^ Student’s *t* test, ^‡^ Mann–Whitney U test, ^#^ Chi-square test, ^§^ Fisher’s exact test, ^¶^ Fisher–Freeman–Halton test.

**Table 2:** Summary of range of motions and assessment scores with regard to groups.

	Groups		
	Control (*n* = 23)	Mulligan (*n* = 23)	*p* (between Groups)
Flexion ROM			
Baseline	47.00 ± 18.26	47.13 ± 16.11	0.965 ^‡^
Post-treatment	58.57 ± 14.39	69.22 ± 7.84	0.006 ^‡^
*p* (within groups)	<0.001 ^¶^	<0.001 ^¶^	
Post-treatment, adjusted (95% CI)	58.60 (55.74–61.46)	69.18 (66.32–72.04)	<0.001 ^§^
Mean difference (95% CI)	10.58 (6.54–14.62)		
Effect size (95% CI)	0.334 (−0.248–0.916)		
Extension ROM			
Baseline	14.43 ± 5.17	14.09 ± 5.11	0.819 ^†^
Post-treatment	18.70 ± 3.27	21.48 ± 2.54	0.002 ^†^
*p* (within groups)	<0.001 ^†^	<0.001 ^†^	
Post-treatment, adjusted (95% CI)	18.61 (18.09–19.13)	21.57 (21.05–22.09)	<0.001 ^#^
Mean difference (95% CI)	2.96 (2.23–3.70)		
Effect size (95% CI)	0.606 (0.015–1.197)		
Right rotation ROM			
Baseline	27.91 ± 8.06	29.00 ± 7.34	0.635 ^†^
Post-treatment	34.83 ± 5.97	41.00 ± 2.68	<0.001 ^†^
*p* (within groups)	<0.001 ^†^	<0.001 ^†^	
Post-treatment, adjusted (95% CI)	35.08 (33.84–36.32)	40.75 (39.50–41.99)	<0.001 ^#^
Mean difference (95% CI)	5.67 (3.91–7.43)		
Effect size (95% CI)	0.495 (−0.092–1.082)		
Left rotation ROM			
Baseline	28.04 ± 7.91	30.39 ± 6.53	0.344 ^‡^
Post-treatment	34.91 ± 6.95	40.87 ± 2.78	0.001 ^‡^
*p* (within groups)	<0.001 ^¶^	<0.001 ^¶^	
Post-treatment, adjusted (95% CI)	35.54 (33.98–37.09)	40.25 (38.69–41.80)	<0.001 ^§^
Mean difference (95% CI)	4.71 (2.50–6.92)		
Effect size (95% CI)	0.317 (−0.265–0.899)		
Right lateral flexion ROM			
Baseline	20.96 ± 5.80	21.70 ± 7.33	0.817^‡^
Post-treatment	26.17 ± 5.21	30.96 ± 3.52	0.001^‡^
*p* (within groups)	<0.001 ^¶^	<0.001 ^¶^	
Post-treatment, adjusted (95% CI)	26.38 (25.31–27.45)	30.75 (29.68–31.82)	<0.001^§^
Mean difference (95% CI)	4.37 (2.86–5.88)		
Effect size (95% CI)	0.536 (−0.052–1.124)		
Left lateral flexion ROM			
Baseline	21.91 ± 5.92	23.74 ± 6.28	0.311 ^‡^
Post-treatment	27.09 ± 5.38	32.48 ± 2.79	<0.001 ^‡^
*p* (within groups)	<0.001 ^¶^	<0.001 ^¶^	
Post-treatment, adjusted (95% CI)	27.58 (26.42–28.75)	31.98 (30.82–33.14)	<0.001 ^§^
Mean difference (95% CI)	4.40 (2.74–6.05)		
Effect size (95% CI)	0.442 (−0.143–1.027)		
Visual Analogue Scale score			
Baseline	6.09 ± 2.11	6.09 ± 1.76	1.000^†^
Post-treatment	4.09 ± 1.90	2.35 ± 1.23	0.001^†^
*p* (within groups)	<0.001 ^†^	<0.001 ^†^	
Post-treatment, adjusted (95% CI)	4.09 (3.73–4.45)	2.35 (1.99–2.71)	<0.001 ^#^
Mean difference (95% CI)	−1.74 (−2.25–−1.23)		
Effect size (95% CI)	0.524 (−0.064–1.112)		
Oswestry Disability Index score			
Baseline	55.65 ± 25.57	54.17 ± 23.74	0.974 ^‡^
Post-treatment	33.83 ± 24.59	15.04 ± 11.89	0.009 ^‡^
*p* (within groups)	<0.001 ^¶^	<0.001 ^¶^	
Post-treatment, adjusted (95% CI)	33.33 (29.16–37.50)	15.54 (11.37–19.71)	<0.001 ^§^
Mean difference (95% CI)	−17.79 (−23.69–−11.89)		
Effect size (95% CI)	0.441 (−0.144–1.026)		

Descriptive statistics were presented by using mean ± standard deviation for actual values and mean (95% confidence interval) for adjusted values. CI: Confidence interval, ROM: Range of motion. ^†^ Two-way repeated measures analysis of variance (ANOVA), ^‡^ Mann–Whitney U test, ^#^ Analysis of covariance (ANCOVA) with Baseline as covariate, ^§^ Quade’s nonparametric analysis of covariance (ANCOVA) with Baseline as covariate, ^¶^ Wilcoxon signed ranks test.

## Data Availability

The data presented in this study are available on request from the corresponding author.
